# Ruptured Abdominal Aortic Aneurysm with a Suprarenal Tumor

**DOI:** 10.21470/1678-9741-2017-0166

**Published:** 2018

**Authors:** Ali Ahmet Arıkan

**Affiliations:** 1 Department of Cardiovascular Surgery, Muş State Hospital, Muş, Turkey.

**Keywords:** Aortic Aneurysm, Abdominal, Aorta/Surgery, Neuroendocrine Tumors, Pheochromocytoma

## Abstract

This paper presents a case study of a patient that underwent surgery for a ruptured abdominal aneurysm. The postoperative course was complicated by resistant hypertension and tachycardia. A suprarenal mass was detected in the computed tomography scan with radiological suspicion of pheochromocytoma. Few cases of pheochromocytoma coexisting with aneurysms have been reported. Management of cardiovascular stability is crucial in such cases. Despite the lack of evidence, pheochromocytomas might have a role in the etiology of aortic aneurysms.

**Table:** 

Abbreviations, acronyms & symbols	
AAA	= Abdominal aortic aneurysm
BMI	= Body mass index
Ca	= Calcium
CT	= Computed tomography
GAS	= Glasgow Aneurysm Score

## INTRODUCTION

Abdominal aortic aneurysms (AAA) are the most common type of aortic aneurysms. Smoking, male sex, age, hypertension, chronic obstructive pulmonary disease, hyperlipidemia and family history are risk factors for this disease. It is often asymptomatic and is diagnosed incidentally. Rupture of an AAA is a catastrophic event with high mortality.

Pheochromocytoma is an unexpected cause of hypertension. As result of catecholamine secretion persistent or intermittent symptoms occur. Despite the lack of evidence, pheochromocytomas might have a role in the etiology of aortic aneurysms. The coexistence of pheochromocytoma and AAA should be carefully managed during surgical treatment considering characteristics of both lesions.

## CASE REPORT

**Fig. 1:**
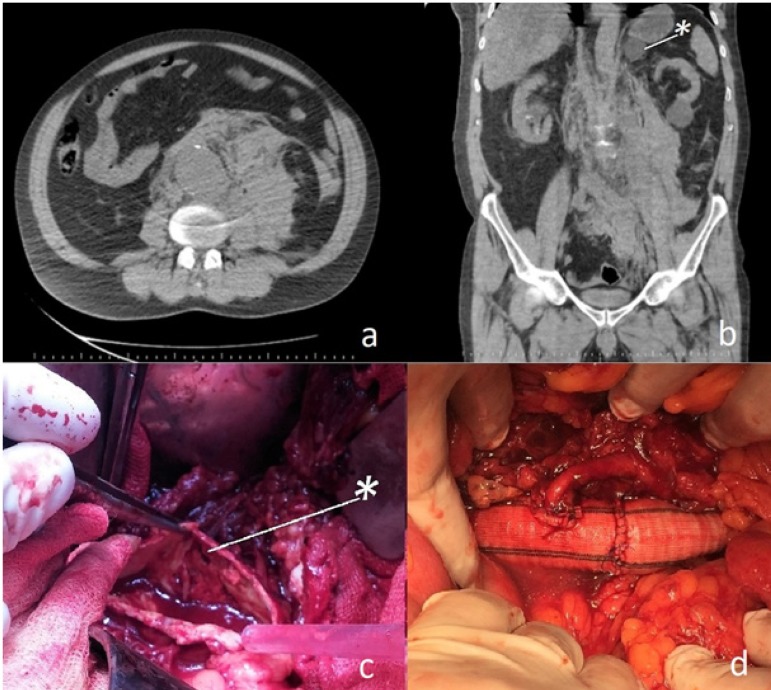
Non-contrast computed tomography images showing a large haematoma on the left retroperitoneal area (a). A homogeneous right suprarenal tumor with radiodensity of 15 Hounsfield units is marked (b). Operative view after aortotomy; the marker shows the tear on the left side of the aneurysm sac (c). Inferior mesenteric artery anastomosis on the tubular graft; a tubular graft was prepared for interposition, but excision of the terminal aorta was required due to its fragile structure. A bifurcated graft was used for distal anastomosis to the iliac arteries (d).

**Fig. 2:**
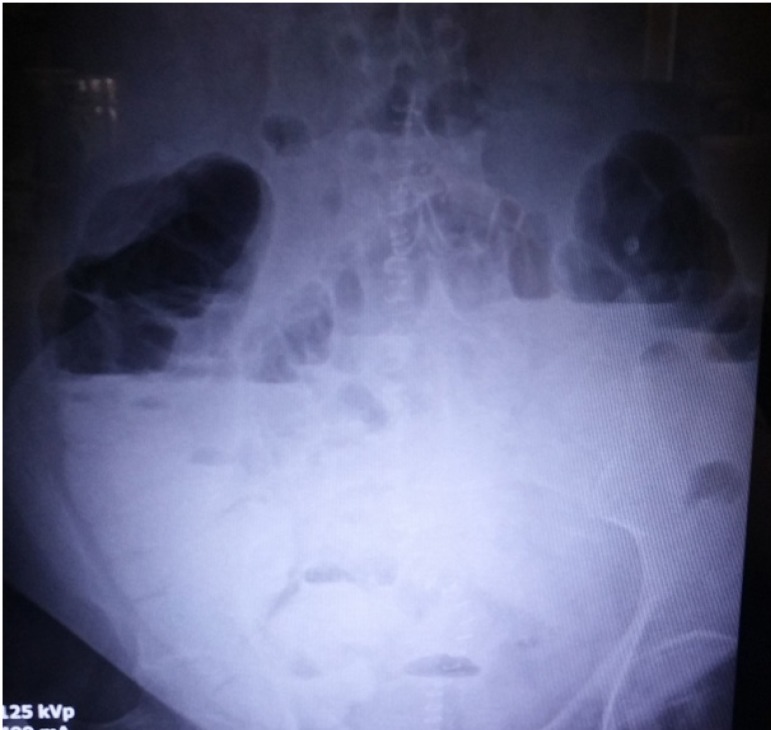
Plain X-ray of the abdomen 8 hours after administration of oral contrast.

A 57-year-old man with hypertension, chronic kidney disease, chronic pulmonary obstructive disease, a 33 cm^2^/m body mass index (BMI), and a history of ureterorenoscopy four years earlier was admitted to the emergency department with flank pain. Renal colic due to urolithiasis was suspected. A computed tomography (CT) scan without contrast revealed multiple renal cysts and two calculi larger than 1 cm on the right renal calix, an aortic aneurysm 57 mm in diameter, and retroperitoneal hematoma (Figures 1A and 1B). The patient's general condition deteriorated, he became hypotensive and lost consciousness. The classical triad of a ruptured AAA is easily identified with the aid of radiological imaging instead of palpation of a pulsatile mass. The patient was immediately transferred to the operating room and aortobiiliac graft replacement was performed with an 18x9 mm bifurcated graft for a ruptured infrarenal AAA. Initially, manual compression at the level of renal artery and vein was performed by the surgical team, and the admission of bolus doses of noradrenalin and crystalloids by the anesthesia team provided enough time to expose the neck of the aneurysm and to perform proximal clamping to the infrarenal aorta. Distally, both iliac arteries were exposed and clamped. After proximal and distal control, 5000 units of heparin were administered intravenously. Longitudinal aortotomy was performed. The aneurysm sac was free of thrombus, and the ostium of the inferior mesenteric artery was patent. A tear was identified on the left side of the aorta (Figure 1C). Our initial strategy to implant a tubular graft was changed based on the need to excise the aortic bifurcation due to its friable tissue. The inferior mesenteric artery without sufficient backflow was replanted (Figure 1D). The patient was transferred to the intensive care unit. He was extubated after 48 hours. Continuous positive airway pressure with a mask was maintained for four days postoperatively. In the postoperative course, the patient underwent several hemodialysis sessions, was oxygen dependent, and experienced paralytic ileus. Hemodialysis was needed daily after postoperative day 2, even though sufficient urine output was present on postoperative day 5. On postoperative day 6, abdominal distension was apparent despite nasogastric decompression. Oral administration of 50 ml of contrast in 500 ml of water was used 8 hours before radiography to evaluate the prolonged postoperative ileus (Figure 2). As there was passage to the colon, we continued to follow up with dexpanthenol 500 mg twice/day, neostigmine 0.5 mg once/day, and an enema twice/day. Oral contrast was administered again on postoperative day 9 to profit from its stimulant effect on intestinal peristalsis^[1]^. The need for hemodialysis and the postoperative ileus resolved 10 days after surgery, and the patient was mobilized without oxygen support. During the postoperative course, the patient was hypertensive, and tachycardia was present. He was on sinus rhythm and oral metoprolol 100 mg twice/day was given to keep the rhythm at 100 beats/min. His blood pressure was controlled with nitroglycerin and alpha (α) and calcium (Ca) channel blockers. We investigated the potential causes of this condition. Two-dimensional echocardiography was performed; the results showed normal ejection fraction and mild tricuspid insufficiency. Re-evaluation of the initial preoperative CT scan revealed a left suprarenal incidentaloma with 4 cm diameter (Figure 1B). The presence of a left suprarenal adenoma on the CT scan with an unenhanced density greater than 10 Hounsfield units resulted in the suspicion that a pheochromocytoma was the etiology of the patient's hypertension^[2]^.

## DISCUSSION

AAA is often asymptomatic. An abdominal aortic diameter greater than 55 mm is an indication of the need for elective surgical or endovascular treatment. Endovascular procedures are commonly performed for treatment, but access to these procedures can be limited depending on a hospital's capabilities. Rupture of an AAA is a catastrophic event with a high mortality rate. In aortic emergencies, the transfer of a patient should be avoided and urgent surgery is indicated^[3]^.

Patients with pheochromocytoma often have hypertension and the triad of palpitation, headache, and sweating. Kota et al.^[4]^ reported that 14% of cases with pheochromocytoma had additional vascular lesions. Few cases of pheochromocytoma coexisting with aneurysms have been reported. Management of cardiovascular stability is crucial in such cases. Optimization of the hemodynamic status can last months in elective cases. An increased risk of rupture of the aneurysm is present due to excess catecholamine and hypertension. Intraoperative and postoperative managements of an aortic aneurysm include strict blood pressure stabilization, which can be challenging in the presence of pheochromocytoma. In a case diagnosed with pheochromocytoma after surgery, dramatic hemodynamic changes were reported during surgery to repair a ruptured AAA^[5]^. In our case, the most apparent hemodynamic change was the persistent hypertension that began after aortic clamping. It reached 160 mmHg, even though the patient was receiving antihypertensive therapy. While a hypothetical association of catecholamine-induced vasculitis or weakening of the vascular wall has been proposed as the reason for the coexistence of pheochromocytoma and AAA^[6]^, evidence to support that claim is insufficient. In our case, the Glasgow Aneurysm Score (GAS) was 88, which is a predictor of high mortality. The patient survived the operation and the early postoperative period of 30 days, and he was referred to a tertiary center for further investigation. In elective cases, excision of the suprarenal tumor followed by a second operation for AAA, or a concomitant surgery for pheochromocytoma and AAA, can be considered. For a ruptured AAA, emergency aortic surgery is indicated; excision of the tumor can be conducted at a later point. During this period, the blood pressure must be managed very carefully. In patients with resistant hypertension and aortic aneurysms, a high index of suspicion is necessary to diagnose suprarenal tumors. Radiologic imaging and assessing the density of a tumor can be helpful for making a diagnosis.

**Table:** 

Authors' roles & responsibilities	
AAA	Substantial contributions to the conception or design of the work; or the acquisition, analysis, or interpretation of data for the work; drafting the work or revising it critically for important intellectual content; final approval of the version to be published
